# Colour Vision Impairment in Young Alcohol Consumers

**DOI:** 10.1371/journal.pone.0140169

**Published:** 2015-10-14

**Authors:** Alódia Brasil, Antônio José O. Castro, Isabelle Christine V. S. Martins, Eliza Maria C. B. Lacerda, Givago S. Souza, Anderson Manoel Herculano, Alexandre Antônio M. Rosa, Anderson R. Rodrigues, Luiz Carlos L. Silveira

**Affiliations:** 1 Instituto de Ciências Biológicas, Universidade Federal do Pará, Belém, Pará, Brazil; 2 Instituto de Ciências da Saúde, Universidade Federal do Pará, Belém, Pará, Brazil; 3 Núcleo de Medicina Tropical, Universidade Federal do Pará, Belém, Pará, Brazil; 4 Hospital Universitário Bettina Ferro de Souza, Universidade Federal do Pará, Belém, Pará, Brazil; 5 Universidade Ceuma, São Luís, Maranhão, Brazil; University of Montreal, CANADA

## Abstract

Alcohol consumption among young adults is widely accepted in modern society and may be the starting point for abusive use of alcohol at later stages of life. Chronic alcohol exposure can lead to visual function impairment. In the present study, we investigated the spatial luminance contrast sensitivity, colour arrangement ability, and colour discrimination thresholds on young adults that weekly consume alcoholic beverages without clinical concerns. Twenty-four young adults were evaluated by an ophthalmologist and performed three psychophysical tests to evaluate their vision functions. We estimated the spatial luminance contrast sensitivity function at 11 spatial frequencies ranging from 0.1 to 30 cycles/degree. No difference in contrast sensitivity was observed comparing alcohol consumers and control subjects. For the evaluation of colour vision, we used the Farnsworth-Munsell 100 hue test (FM 100 test) to test subject’s ability to perform a colour arrangement task and the Mollon-Reffin test (MR test) to measure subject’s colour discrimination thresholds. Alcohol consumers made more mistakes than controls in the FM100 test, and their mistakes were diffusely distributed in the FM colour space without any colour axis preference. Alcohol consumers also performed worse than controls in the MR test and had higher colour discrimination thresholds compared to controls around three different reference points of a perceptually homogeneous colour space, the CIE 1976 chromaticity diagram. There was no colour axis preference in the threshold elevation observed among alcoholic subjects. Young adult weekly alcohol consumers showed subclinical colour vision losses with preservation of spatial luminance contrast sensitivity. Adolescence and young adult age are periods of important neurological development and alcohol exposure during this period of life might be responsible for deficits in visual functions, especially colour vision that is very sensitive to neurotoxicants.

## Introduction

In accordance to World Health Organization (WHO), alcohol is the oldest and widest used psychoactive substance consumed by humans around the world [1]. Several works previously described that the alcohol consumption behaviour was spreading among young people living in developing countries [2–5]. It was well documented that precocious alcohol consumption could lead to chemical dependence in both young and adult subjects [6–9]. Besides socio-economic prejudice, alcohol consumption may cause several physiological dysfunctions that very often comprise the impairment of visual functions [1, 10–20].

At the same time that human studies pointed to an impairment of visual functions caused by alcohol toxicity, several studies using animal models showed deleterious effects of alcohol exposure on the visual system [21–23]. Kjellström and colleagues analysed the VECP recorded from adult rats exposed to ethanol [21]. In ethanol-exposed rats examined without withdrawal, the VECP showed an increase in onset latency and a marked distortion of the N1 component, alterations that were partly normalized one week after withdrawal [21]. Sancho-Tello and colleagues showed that chronic ethanol consumption induced oxidative stress in the rat retina, ERG alterations, and overexpression of the antiapoptotic Bcl-2 protein, and suggested that these pathological findings could be explained by an alcoholic retinopathy in this animal model [22]. Lantz and colleagues tested whether early alcohol exposure would lead to visual function impairment in mice by recording the electroretinogram (ERG), visual evoked cortical potential (VECP), and visual cortex optical imaging [23]. They found that mice exposed to ethanol, in comparison with controls, displayed similar spatial frequency acuity, lower contrast sensitivity, ERG with smaller a- and b-waves, and distorted visual cortex retinotopic maps [23].

The mechanisms of alcohol toxicity on the visual system are not fully understood. However, there are evidences that a variety of neurotransmitter systems are affected by ethanol [24–26]. Several evidences supported the idea that ethanol may cause many of its effect by modulating ligand-gated ion channels responsive to GABA, glutamate, and serotonin neurotransmitters [27–28]. Results demonstrating that enhancers of GABA activity were effective in the management of ethanol withdraw suggested that the GABAergic system is an important target for alcohol toxicity [29]. In addition, decreased levels of GABA(A)-benzodiazepine receptor complex in the brain of chronic alcoholic subjects gives support to the hypothesis that ethanol and GABA interplay modulates a central nervous system dysfunction in such subjects [30].

Previous psychophysical evaluations described visual field and spatial luminance contrast sensitivity losses in chronic alcohol consumers [16] and after acute alcohol consumption [13, 17, 20]. Classical studies using Farnsworth-Munsell 28-hue or 100-hue color-arrangement tests showed high incidence of colour vision losses in chronic alcohol consumers [10, 15]. In addition, it was demonstrated that subjects with history of alcoholism exhibit colour vision changes without impairment of spatial luminance contrast sensitivity [18]. Several studies tried to characterize specific visual deficits associated with alcohol consumption, but the results were contradictory [16, 18]. Thus, considering the social impact of this issue, it is very important to intensify studies aiming a detailed characterization of visual dysfunctions in adult or young people acutely or chronically exposed to alcohol.

Few studies described the effect of precocious alcohol consumption on the visual perception of young people. Previous reports demonstrated that exposure to different xenobiotics such as ethanol [18, 31], methanol [32–33], chloroquine and hydroxichloroquine [34–36], organic solvents [37], and heavy metals such as mercury compounds [38–43], causes pronounced alterations of visual functions. Some of these studies have specifically focused exposure at early ages [44]. Toxicological studies performed in humans and animals showed that immature or young subjects have higher sensitivity to toxicants than adults [45–47].

These studies support our hypothesis that young consumers that are frequently exposed to ethanol may show subclinical visual alterations that could be revealed with appropriate psychophysical evaluation. Psychophysics comprises important perceptual and non-invasive tests commonly used to verify the normal or abnormal functionality of human senses, including vision [18, 34, 42, 48–50]. In this way, the aim of the present study was to evaluate chromatic and achromatic visual functions of young alcohol consumers with three widely used psychophysical tests–measurement of spatial luminance contrast sensitivity, evaluation of the colour arrangement ability, and measurement of colour discrimination thresholds.

## Materials and Methods

### Subjects

We studied 24 young alcohol consumers, 17 men and 7 women, with age between 18 and 29 years old (22.6 ± 3.7 years old). Some years ago, our research group divided subjects per age groups for a series of studies on visual dysfunctions in the following ranges: 16–30 years old, 31–45 years old, 46–60 years old, and 61–75 years old. This was largely influenced by the following arguments: i) the first group pivoted around the age of young adults – 24 years old; ii) the second group comprised mature adults; iii) the third and fourth groups comprised two groups of elderly people. This systematic allowed a readily comparison between results obtained in different studies, especially those aimed to understand the effects of diseases and xenobiotics on colour vision and spatial vision [42].

All subjects consumed alcoholic beverages at least twice per month and 5 of them also smoked cigarettes (10–20 per day). The psychophysical tests were performed at least 48 hours after the last alcohol consumption. All subjects had no history of past or present ocular or neural diseases or chemical exposure that could affect the visual functions. The results of the young alcohol-consumers were compared with population norms derived from healthy volunteers with no history of alcohol consumption, age ranging between 16 and 30 years old (see below in the Results section for further details of the control group). All subjects answered a questionnaire comprising the following information: age that they started taking alcoholic beverages; monthly frequency of alcohol intake; amount of alcohol intake in a single day; and the association of alcohol consumption with smoking-behaviour.

All subjects were examined by an ophthalmologist before psychophysical testing and were considered normal results in the ophthalmological evaluation. All subjects were additionally investigated to select them against congenital colour blindness either red-green or blue-yellow. This was performed using the following strategy. First, during the anamnesis they were inquired about colour blindness incidence among their relatives and also any difficult with colours in their own childhood. Second, the subjects’ colour vision was tested with the Ishihara Pseudoisochromatic plates applied separately to each eye [51]. Third, we inspected subjects’ results in the MR test (see below) to see whether they presented colour vision thresholds elevated only along lines oriented towards the protan, deutan, and tritan copunctual points. Some subjects that volunteered to be part of the control group were excluded after failed in these criteria. No subjects that volunteered to be part of the alcohol consumers group failed in any of these criteria.

### Ethics statement

This work was approved by the Ethics Committee for Research with Human Subjects of the Tropical Medicine Nucleus, Federal University of Pará, Belém, Pará, Brazil; protocol #038/2004 –CEP / NMT, date of approval 30th April 2004. All subjects signed an informed consent prior to the experiments, which followed The Code of Ethics of the World Medical Association (Declaration of Helsinki) for experiments involving humans.

### Psychophysical procedures

Subjects performed three different tests developed to be used with computers: measurement of spatial luminance contrast thresholds with sinusoidal gratings [52], evaluation of the colour arrangement ability with the Farnsworth-Munsell 100-hue test (FM 100 test) [53–54], and measurement of colour discrimination thresholds with the Mollon-Reffin test (MR test) [55–56]. All subjects had normal or corrected to normal 20/20 visual acuity; spectacle lens were used to correct visual acuity when necessary during the tests. All tests were performed under monocular vision using the eye with best, uncorrected visual acuity or the dominant eye when both eyes provided the same visual acuity. All tests were performed in a dark room with 0.02 cd/m^2^ of background luminance.

Software was written in C++ computer language for IBM RISC 6000 320H workstation (International Business Machine Corporation–IBM, Armonk, New York, USA). A graphical card IBM POWER GT4-3D with colour resolution of 24 bits / 8 bits per gun was used to generate the visual stimuli which were shown in a colour monitor IBM 6091 19i, with spatial resolution of 1280 x 1024 pixels, sampling rate of 77 Hz. A dithering routine was used to obtain 10-bits grey level resolution. Luminance and chromaticity coordinates were measured with a CS-100A chroma meter (Konica Minolta, Mahwah, New Jersey, USA).

The spatial luminance contrast sensitivity was estimated at eleven spatial frequencies (0.2, 0.5, 0.8, 1, 2, 4, 6, 10, 15, 20, and 30 cycles / degree of visual angle) [39, 42]. The stimuli were vertical, stationary, sinusoidal luminance gratings with constant mean chromaticity (CIE1976: u’ = 0.182, v’ = 0.474), mean luminance (43.5 cd/m^2^), size (6.5° x 5° of visual angle), and viewing distance (3 m). The method of adjustment was used; the researcher decrease or increase the spatial luminance contrast and a sound indicated to the subject that he had to report if the grating stimulus was perceptible or not; the procedure was repeated until the luminance threshold for that spatial frequency was reached. The procedure was repeated 6 times for every spatial frequency. The analysis consisted in averaging the 6 results and to use the mean value as representative of the contrast threshold. The contrast sensitivity was expressed as the log of the inverse of the contrast threshold.

A computerized version of the FM 100 test was used to evaluate subject’s ability to perform a hue arrangement [39, 42]. Eighty-five stimuli of different hues, having the same saturation (30% purity), luminance (42 cd/m^2^), shape and size (square patches of 1° of visual angle), were grouped in 1 series of 22 and 4 series of 21 stimuli. Initially, the patches were shown in a gradient of hues that represented the correct ordering sequence. After 1 min inspection, they were randomized in the computer screen and the subject had to reorganize them in the original sequence using the hue variation as the only clue. Once the subject had finished a series of hues, the next series was presented until the subject ordered all the four series. In the end of the test, the number of mistakes made by the subject was saved. The entire test was repeated four times. The software estimated subject’s score for each trial and the average score comprising the four trials was taken as subject score in the hue arrangement task, the higher the score, the worst was the subject performance.

The Mollon-Reffin method was used to measure subject’s colour discrimination thresholds [55–56]. Custom made software was used to provide the Mollon-Reffin test [39, 42]. The pseudoisochromatic stimuli comprised a mosaic of circles with different sizes (0.2 to 0.6° of diameter) and different luminance (12 to 20 cd/m^2^) that formed target and field embedded in a dark background (0.02 cd/m^2^). Circles formed the stimulus field with constant chromaticity throughout a series of measurements while circles whose chromaticity changed from trial to trial formed the target. The stimulus target was a Landolt’s C measuring 4.3° of outer diameter, 2.2° of inner diameter, and with a gap of 1° of visual angle. The target chromaticity varied along 8 chromatic axes radiating from the field reference chromaticity. Five measurements of colour discrimination thresholds were made around five different reference chromaticies in the CIE1976 colour space: E1 (u’ = 0.215, v’ = 0.531), E2 (u’ = 0.219, v’ = 0.481), E3 (u’ = 0.225, v’ = 0.415), E4 (u’ = 0.175, v’ = 0.485), and E5 (u’ = 0.278, v’ = 0.472). Stimuli had mean luminance of 16 cd/m^2^ and were shown for 3 s. The subject’s task was to identify the target orientation (up, down, right, or left positions of the Landolt’s C gap) in the following 1.5 s. The presence of spatial and luminance noise assured that discrimination between target and field was performed based solely in the chromaticity difference. The chromatic difference between target and field was controlled by a four alternative forced-choice staircase procedure: a right response by the subject decreased the chromatic difference, while a wrong response or no response increased the chromatic difference. Twelve reversals stopped the staircase procedure and the average of the last 6 reversals was used to estimate the colour discrimination threshold along each chromatic axis. Ellipse functions were fitted to the colour discrimination thresholds using the least square method. Ellipses’ sizes (diameters of circles with equivalent areas) and orientations were estimated and used as indicators of the subject’s colour discrimination performance.

### Statistical analysis

Data from each young alcohol consumers were compared with tolerance intervals for the control group. The proportion of subjects with results out of the tolerance intervals was taken into account as indicative of visual impairment due to alcohol abuse in this group of young alcohol consumers. In addition, we compared the performance of the two subject groups, young alcohol consumers and controls, in each visual test, using Student t test for FM 100 data and two-way ANOVA for MR data and spatial luminance contrast sensitivity data. For all statistical procedure we considered an alpha value of 5%.

**Table 1:** Epidemiological profile of the young alcohol consumers studied in this work (n = 24).

Subject	Sex	Age (years)	Alcohol consumption (years)	Rate (per month)	Quantity (g)	Smoking	Obs
S01090617	M	20	7	8	Nd	No	1,2
S02100805	F	22	4	4	97	Yes	
S03090813	F	20	2	8	120	Yes	
S04090911	F	21	5	2	42	No	
S05100811	M	27	5	5	98	No	
S06100818	F	21	6	12	140	No	
S07100114	M	25	6	12	360	Yes	
S08100807	M	24	6	8	168	No	
S09090908	M	29	12	4	Nd	No	
S10090617	M	19	2	4	160	No	2
S11090805	F	20	2	3	98	No	
S12101022	F	23	6	8	216	No	
S13091023	M	21	2	2	112	No	
S14091030	M	23	4	3	112	No	
S15100325	M	23	5	8	144	Yes	
S16090716	M	23	2	3	60	No	
S17100730	M	25	6	3	160	No	
S18100804	M	26	7	4	70	No	1
S19100816	M	26	10	8	120	Yes	
S20100219	M	22	2	2	84	No	1,2
S21100222	M	23	10	2	70	No	
S22090709	M	22	5	4	112	No	
S23100818	F	19	2	6	140	No	
S24090708	M	18	2	12	350	No	
**Mean**	** **	**22.6**	**5**	**5.6**	**137.9**	** **	
**S.D.**	** **	**2.7**	**2.8**	**3.3**	**80.8**	** **	

Obs: nd, not declared; smokers made use of 5–10 cigarettes per day

1, subject was unavailable to perform the spatial luminance contrast sensitivity evaluation

2, subject was unavailable to perform the MR test.

**Fig 1:**
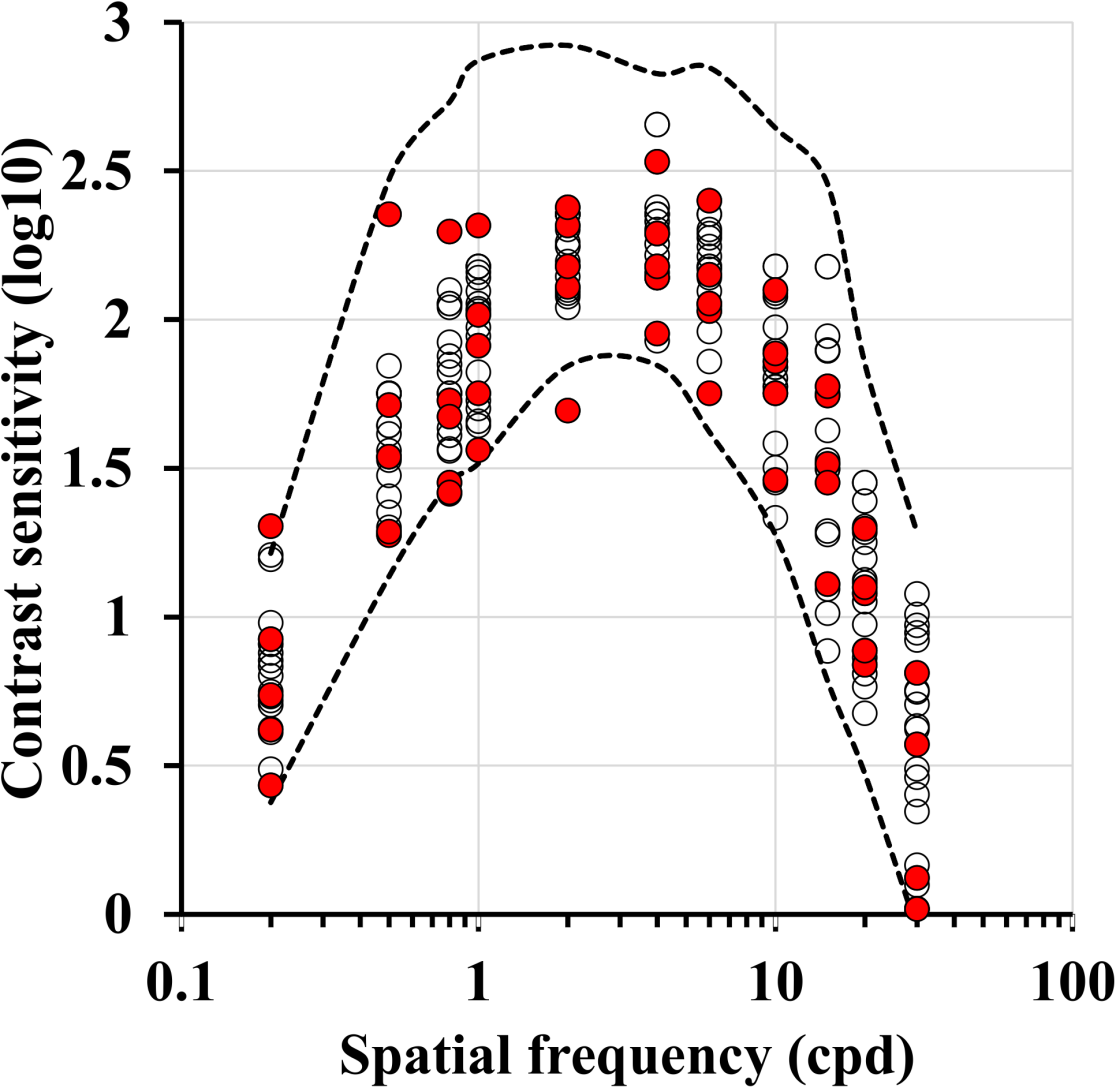
Spatial luminance contrast sensitivity of young alcohol consumers compared with tolerance limits for the control group. Young alcohol consumers had contrast sensitivity in the normal range. Data from alcohol consumers (circles) are located in the tolerance interval (dashed red lines) with the exception of a single data point from one subject that had contrast sensitivity lower than the lower tolerance limit a 2 cpd. Smokers and non-smokers among the alcohol consumers are identified with red and empty circles, respectively. There were no obvious differences between the two groups that could indicate an aggravation of the psychophysical performance of alcohol consumers by the smoking habit.

There were no smokers in the control group. In addition, the number of alcohol consumers that were also smokers was too small (n = 5) in relation to the total of alcohol consumers (n = 19) for a proper statistical comparison between these two groups. However, we identified the smokers in the results presented in the Figs 1, 3 and 5 to appreciate if this additional toxicant could have aggravated subjects’ performance in the psychophysical tests.

We also used Pearson correlation coefficient to measure the linear correlation between the results of spatial luminance contrast sensitivity measurements, FM 100 test, and MR test with indicators of alcohol consumption–years of alcohol consumption, rate of alcohol consumption, and quantity of alcohol consumption.

## Results

### Alcohol consumption

Table 1 lists the information provided by the subjects during their interview. Subjects started alcohol consumption between 13 and 22 years old (17.6 ± 2.1 years old). When they were tested, they had already embarked in routine alcohol consumption for 2–12 years (5 ± 2.8 years) at a frequency between 2–12 times per month (5.6 ± 3.3 times per month). The amount of alcohol consumed was estimated to be between 42 and 360 g / day (137.9 ± 80.8 g / day). Finally, five subjects reported that they also had the habit of smoking between 5–10 cigarettes per day.

### Spatial luminance contrast sensitivity

Three subjects were unavailable to perform the spatial luminance contrast sensitivity (Table 1). Fig 1 shows the contrast sensitivity of the young alcohol consumers (circles, n = 21) and tolerance limits for the control group (dashed red lines) at eleven spatial frequencies studied. Control group for this test comprised 59 healthy volunteers with no history of alcohol consumption or cigarette smoking, 21.2 ± 2.7 years old. With a single exception, the majority of young alcohol consumers had contrast sensitivity inside the tolerance limits at all spatial frequencies; one subject had contrast sensitivity lower than the lower tolerance limit at 2 cpd. The comparison between groups found no statistical differences between young alcohol consumers and controls (P > 0.05, two-way ANOVA).

### Farnsworth-Munsell 100 hue test

**Fig 2:**
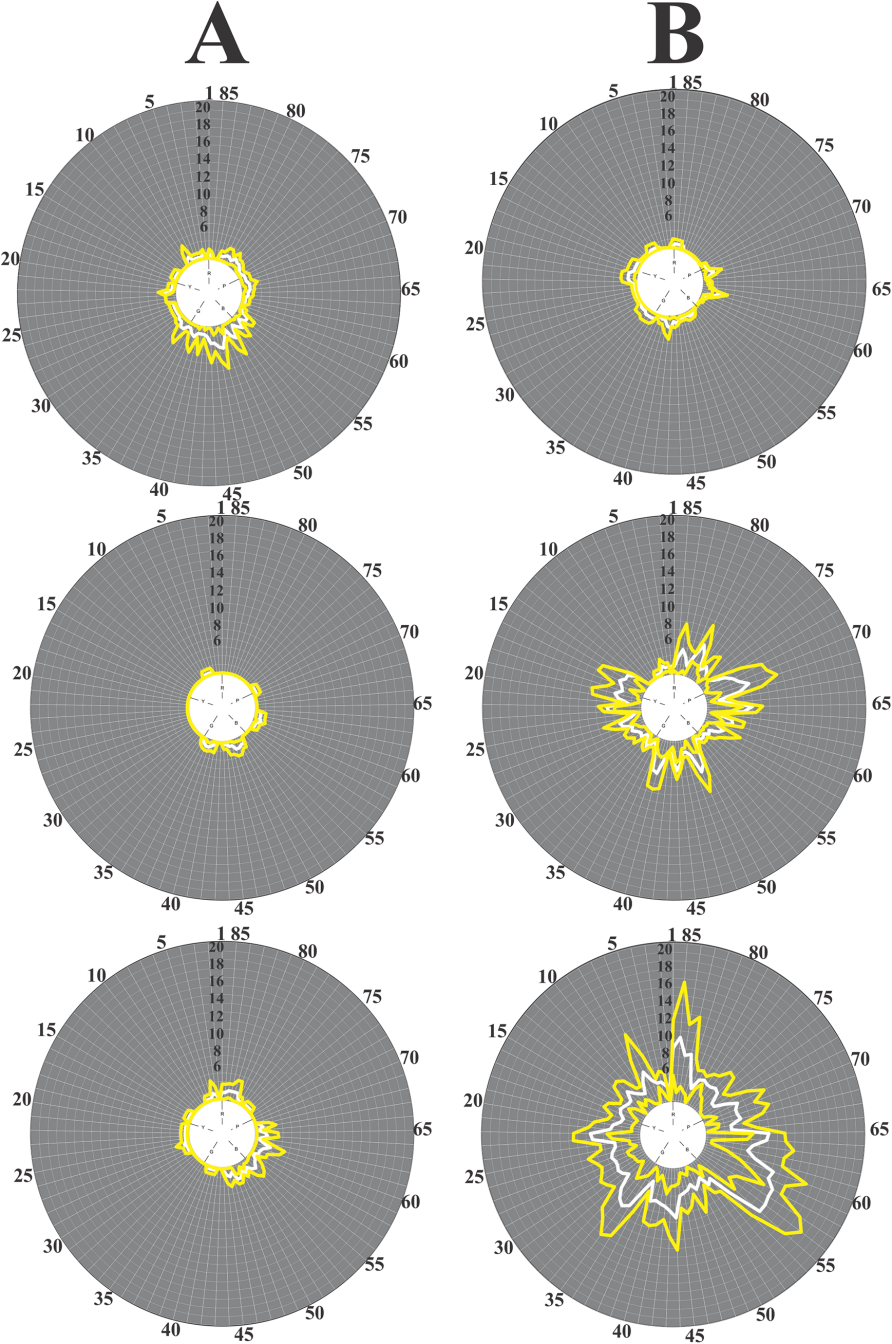
Colour arrangement performance of young alcohol consumers in the FM 100 test compared with performance of young non-alcohol consumers. Examples of individual performance. **A**) Results for three young non-alcohol consumers. Subjects from this group always had small total scores. Top to bottom: subjects C01020222 (right eye, 21 years old), C02000111 (right eye, 20 years old), and C03010429 (right eye, 18 years old) had total scores of 53 ± 19, 13 ± 7, and 36 ± 9, respectively. **Β**) Results for three young alcohol consumers. Some subjects from this group performed similarly to controls in this test while others made more mistakes than predicted by the upper tolerance limits for controls. Top to bottom: subjects S15100325 (Right Eye, 23 years old), S08100807 (Right Eye, 24 years old), and S01090617 (Right Eye, 20 years old) had total scores of 28 ± 3, 152 ± 10, and 326 ± 30, respectively. See text for additional details.

All subjects performed this test (Table 1). Fig 2 shows results of hue arrangement performed by three control subjects (Fig 2A) and three young alcohol consumers (Fig 2B). They were chosen to represent the range of variation observed in the two groups. The plots illustrate means (white contours) and standard deviations (yellow contours) for the score of mistakes made by a subject (n = 4 trials). Data were plotted in the Farnsworth-Munsell polar diagram where different radial directions represent hues and distances from the centre represent score for mistakes made by the subject in the hue arrangement. The inner white circle represents a score of 2 at all 85 directions, meaning that the subject made no mistake and all the hues were placed in the correct order. We quantified subject performance by estimating the total error as the sum of all individual scores for different hue directions minus 170. A total error of zero corresponded to no mistakes made by the subject. Young non-alcohol consumers had small total scores (Fig 2A) while young alcohol consumers had a range of total errors varying from values in the normal range to much higher values (Fig 2B). Young alcohol consumers that had high values of total errors showed no preference for any specific direction in the FM diagram once their mistakes had a diffuse distribution in the colour space.

**Fig 3:**
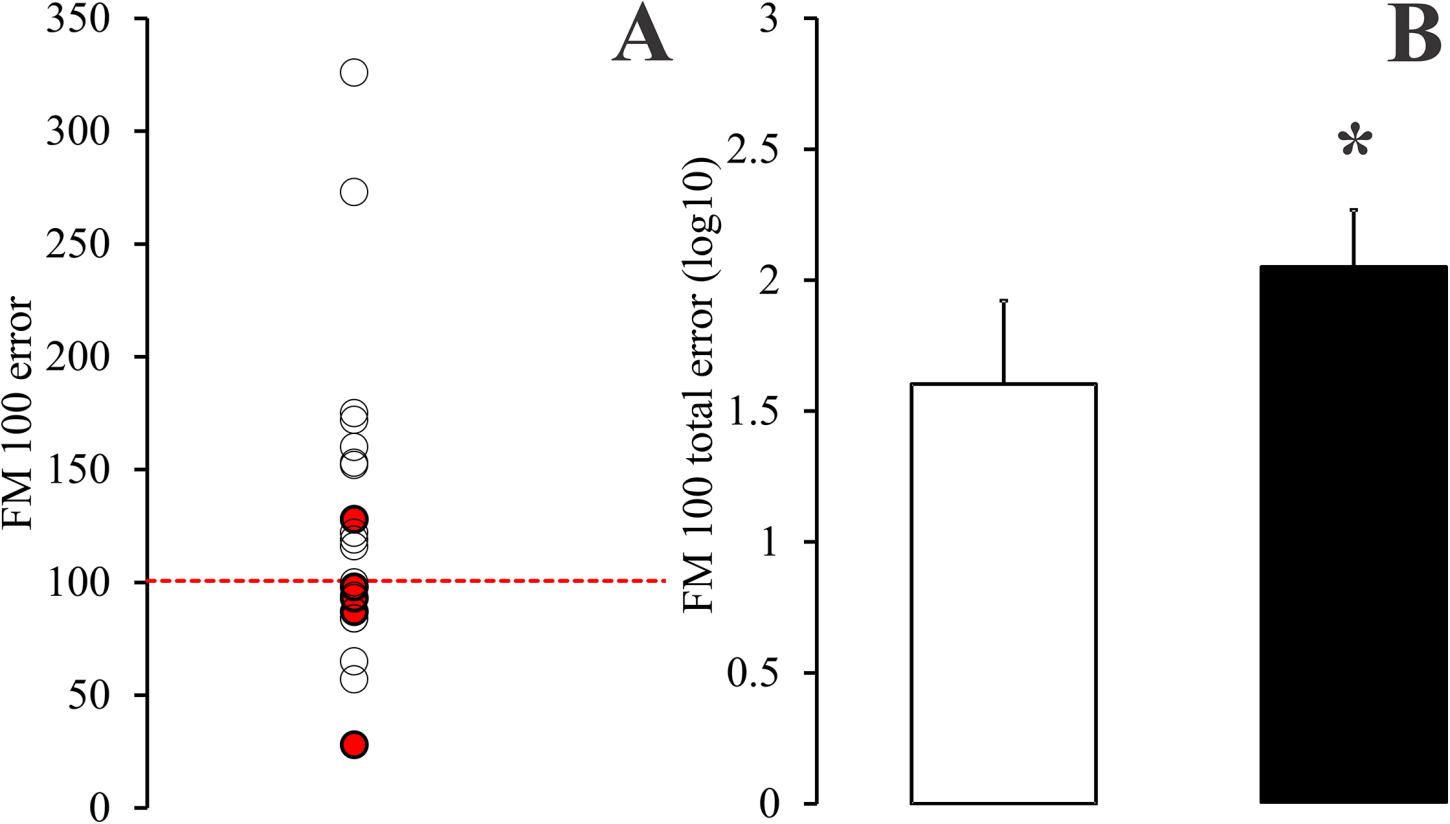
Colour arrangement performance of young alcohol consumers in the FM 100 test compared with performance of young non-alcohol consumers. Results for the two groups. **A**) Total errors for young alcohol consumers compared with the upper tolerance limit for young non-alcohol consumers. Half of alcohol consumers made more mistakes than the upper tolerance limit estimated for the control group, 100.7. Smokers and non-smokers among the alcohol consumers are identified with red and empty circles, respectively. There were no obvious differences between the two groups that could indicate an aggravation of subjects’ performance in the hue arrangement test by the smoking habit. **Β**) Comparison between means and standard deviations for controls (empty column) and alcohol consumers (filled column) (log values). Alcohol consumers had higher total errors than controls; their results were also more variable. (*) Statistical significance (P < 0.01, Student t test).

Fig 3A shows the distribution of values for total errors in the FM 100 test for young alcohol consumers (circles, n = 24) compared with the upper tolerance limit for controls of the same age range (dashed line). Control group for this test comprised 83 healthy volunteers with no history of alcohol consumption, 20.8 ± 2.8 years old. The young alcohol-consumers had total errors ranging from 28 to 326, half of them above the upper tolerance limit for controls (100.7). Fig 3B shows total error means and standard deviations for young non-alcohol consumers (empty column) and young alcohol consumers (filled column) (log values). When we compared the log values of total errors for the two groups, we found that young alcohol consumers made significantly more mistakes than controls in the colour arrangement test, 2.05 ± 0.22 versus 1.6 ± 0.32 (P < 0.01, Student t test).

### Colour discrimination thresholds (MacAdam colour discrimination ellipses)

**Fig 4:**
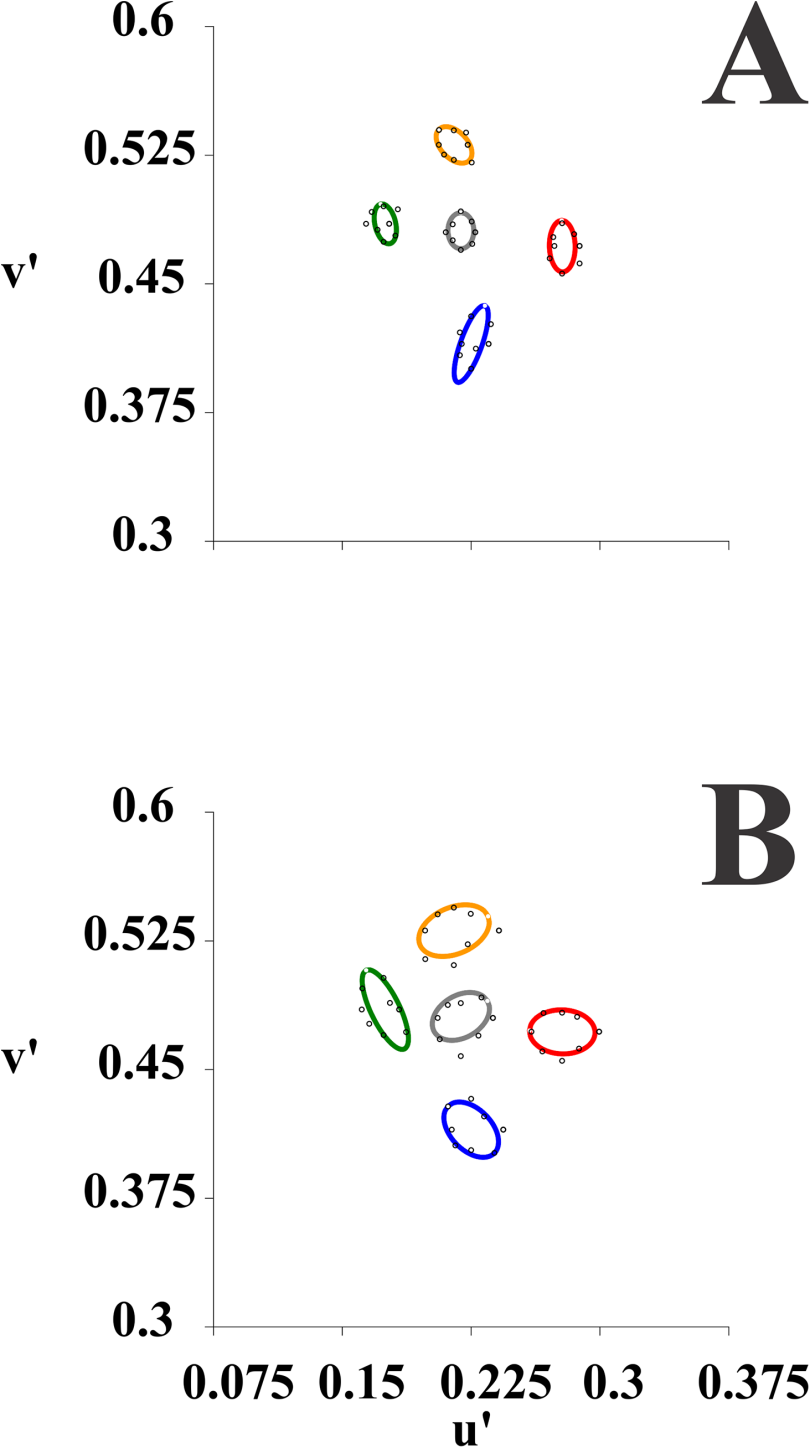
Colour discrimination ellipses from young alcohol consumers obtained with the MR test compared with young non-alcohol consumers. Examples of individual performance. Five regions of the colour space were studied. Data points from colour discrimination thresholds were plotted in the CIE 1976 colour space and then fitted to obtain the corresponding MacAdam colour discrimination ellipses. **A**) Colour discrimination ellipses obtained from a control subject (C04 020620 Right Eye, 20 years old). **Β**) Colour discrimination ellipses obtained from a young alcohol consumer (S02100805, right eye, 22 years old). Some alcohol consumers had colour discrimination ellipses larger than controls, but data for the majority of them fell in the normal range. There was no preference in the colour space for threshold increase in young consumers.

Three subjects were unavailable to perform the MR test (Table 1). We estimated the colour discrimination ellipses in 5 different regions of the CIE1976 colour space for young alcohol consumers (n = 21) and compared the results with those obtained from controls. Control group for this test comprised 51 healthy volunteers with no history of alcohol consumption, 20.8 ± 3.1 years old. Fig 4A shows the colour discrimination ellipses obtained from a control subject, while Fig 4B is from a young alcohol consumer. This alcohol consumer and a few others had colour discrimination ellipses larger than controls. Threshold increase in alcohol consumers had a diffuse nature with no preferential direction in the colour space.

**Fig 5:**
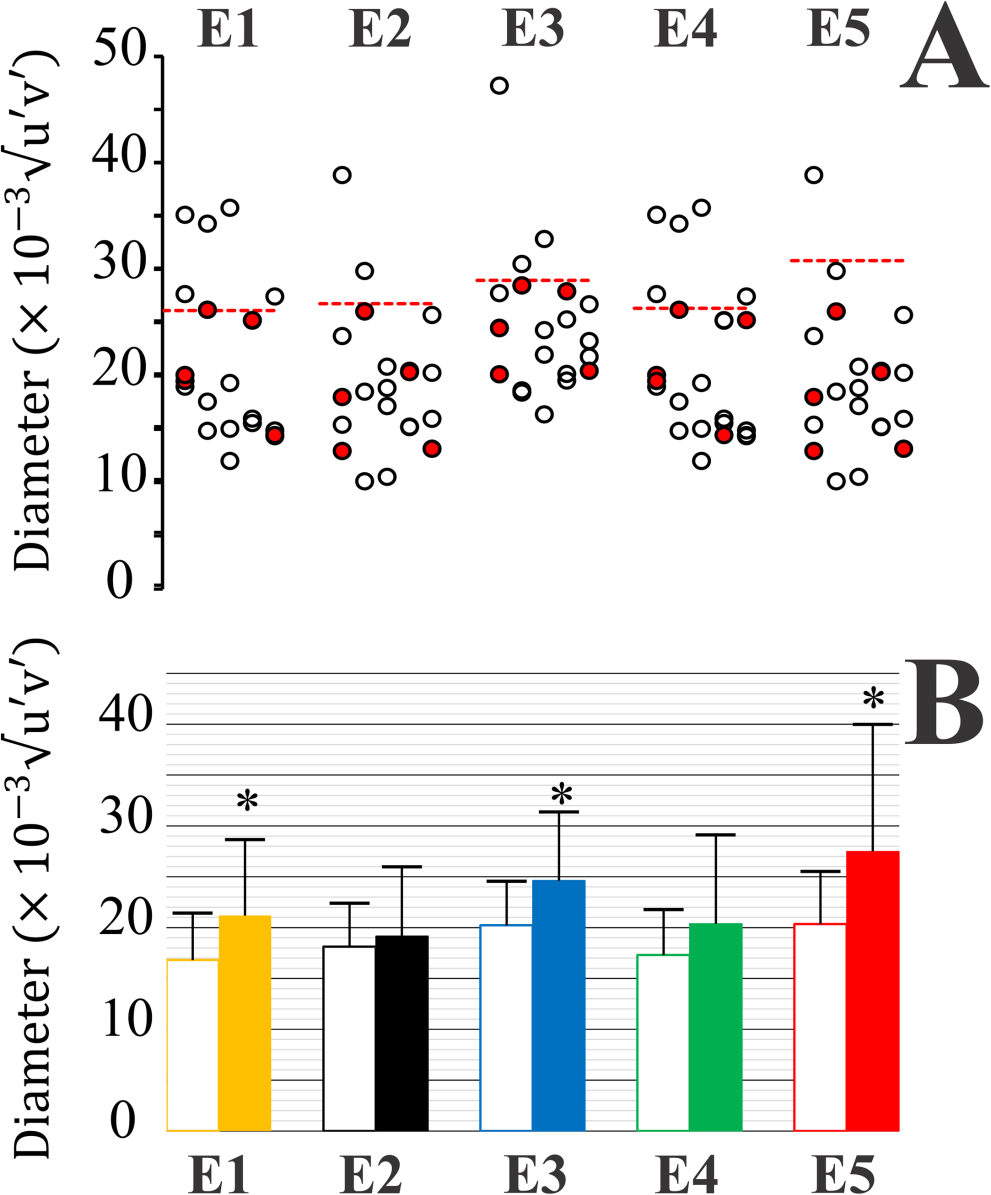
Colour discrimination ellipses from young alcohol consumers obtained with the MR test compared with young non-alcohol consumers. Results for the two groups. **A**) Diameter of colour discrimination ellipses obtained from young alcohol consumers (circles) compared with upper tolerance limits for controls. Ellipse diameter was taken as the diameter of a circle with equivalent area. Depending on the ellipse, E1 to E5, between 1 and 5 subjects had ellipses larger than the upper tolerance limits. Smokers and non-smokers among the alcohol consumers are identified with red and empty circles, respectively. There were no obvious differences between the two groups that could indicate an increase in colour discrimination thresholds of alcohol consumers by the smoking habit. **Β**) Means and standard deviation for ellipse diameters obtained from young alcohol consumers (filled bars) and controls (empty bars). For all ellipses, E1 to E5, the group of alcohol consumers had larger ellipses than the group of non-alcohol consumers, but statistical significance was reached only for E1, E3, and E5 (P < 0.05, ANOVA).

Fig 5 compares the performance of young alcohol consumers and young non-alcohol consumers in the MR test, using the sizes of five colour discrimination ellipses as comparative parameters. Ellipse size was taken as the diameter of the circle with equivalent size measured in the CIE 1976 coordinates. Ellipses E1 to E5 correspond to different regions of the CIE colour space (see Fig 4). Fig 5A shows individual results for alcohol consumers (circles) compared with the upper tolerance limits for non-alcohol consumers. Depending on the ellipse, 1 to 5 subjects had ellipses larger than the upper tolerance limit: 5 subjects had larger ellipses than the upper tolerance limits for E1 and E4. Fig 5B shows means and standard deviations of ellipse areas for young alcohol consumers (filled bars) compared with young non-alcohol consumers (empty bars). For all ellipses, E1 to E5, the group of alcohol consumers had larger ellipses than the group of non-alcohol consumers, but statistical significance was reached only for E1, E3, and E5 (P < 0.05, ANOVA).

### Additional statistical comparisons

Pearson correlation coefficient measurements did not show any significant correlation between log of luminance contrast sensitivity at any spatial frequency, log FM 100 total error, or MR ellipse diameters with indicators of alcohol consumption–years of alcohol consumption, rate of alcohol consumption, and quantity of alcohol consumption (p > 0.05).

## Discussion

The marketing of companies of alcoholic beverages usually encourages alcohol consumption among young adults, targeting this parcel of the population [57]. Alcohol consumption limited to weekends is socially accepted and of little public health concern. This study showed that young adults with limited, weekly habits of alcohol consumption already exhibit signs of visual function impairment when compared to control subjects of the same age.

Zrenner and colleagues previously described that young trichromats exhibited colour vision impairment between 60 and 130 min after ethanol consumption [31]. These authors suggested that acute ethanol exposure was able to induce fast and dose-dependent impairment of colour arrangement performance in young people. In another study, Mergler and colleagues described that both age and alcohol consumption may cause dyschromatopsia [12]. However, no studies investigated whether colour vision of adolescents and young adults was more vulnerable to ethanol toxicity than other visual functions such as luminance spatial contrast sensitivity. In the present study, we showed that young moderate alcohol consumers with normal luminance spatial contrast sensitivity exhibited colour vision impairment even without acute alcohol exposure. Our results contribute to the toxicology of human alcohol exposure by demonstrating that low frequency of alcohol consumption induced significant sustained deleterious effect on the colour vision of young alcohol consumers.

We found that some aspects of colour vision such as the ability to perform the arrangement of series of different hues and the colour discrimination thresholds were affected in sporadic young alcohol consumers. Although previous studies described a profound effect of alcohol consumption on visual functions of adults with prolonged chronic exposure to ethanol [10–18, 20], the present study was the first to demonstrate that young people with low frequency of alcohol consumption exhibit significant colour vision impairment. This study also demonstrated that colour vision was more vulnerable to the alcohol effect than luminance spatial contrast sensitivity in young people. Our results showed that hue arrangement ability and colour discrimination thresholds were impaired in a significant number of alcohol consumers when compared with controls, while spatial luminance contrast sensitivity function was similar in the two groups. Thus, our data suggest that psychophysical evaluation of colour vision function may represent an important tool for the early diagnostics and monitoring of ethanol toxicity in young consumers.

Our psychophysical evaluation also demonstrated that colour vision impairment had no specific preference for chromatic axes. Previous studies reported that colour vision deficits associated with alcohol consumption occurred predominantly along the blue-yellow axes [12]. It was proposed that S cones would be more susceptible to alcohol toxicity than L or M cones. We have not observed such preference in our study and this might be due to the amount of alcohol consumption and / or the younger age of subjects that we studied.

Independently of chromatic axes preference, colour vision was more susceptible to toxic exposure than luminance spatial contrast sensitivity. Human colour vision is more pronounced in the central vision where cell density is very high and metabolic provision could be a problem under toxicant aggression. In the retina, colour vision is supported by the numerous red-green colour-opponent P cells and blue-yellow small bistratified cells that reached the highest cell density in the central region [58]. In the visual cortex, chromatic information is processed by neurons located in layer 4Cb and compartments called “blobs” of layers 2–3 of V1, as well as in the thin strips of V2 [59–60], which have high metabolic activity and are rich in enzymatic activity such as cytochrome oxidase. These enzymes have important role in the mitochondria cellular respiratory electron transport chain and, for instance, neurons located in the blobs exhibit high-energy consumption [61]. As the alcohol can decrease the intracellular storage of ATP [62], these cells also are susceptible to energetic deficits. It is well documented that decreased levels of ATP is closely associated to overproduction of reactive oxygen species (ROS) and that ROS production is an important intermediary of alcohol toxicity in the central nervous system [63–64]. Our findings open new perspectives for the evaluation of cellular or molecular mechanisms supporting colour vision function during alcohol exposure in young adults.

This work reports that low-to-moderate consumption of alcohol is detrimental to colour discrimination thresholds and the ability to perform colour arrangements. These findings seem to be in discrepancy with other reports suggesting that low-to-moderate ethanol consumption is protective to other brain functions and other organic functions. The alcohol consumption of low-to-moderate doses is associated to a decreased risk of cardiovascular diseases [65]. Part of the protective cardiovascular effect of alcohol consumption is associated to the increase of HDL cholesterol and decrease of fibrinogen and other thrombotic factors [65–66]. As many organic and neural diseases share common risk factors with or are secondary to cardiovascular diseases, low-to-moderate alcohol consumption could have a direct or secondary protective effect, decreasing the risk of acquire these diseases and diminishing the impact that they may have on some higher nervous functions such as cognition and memory [67–71].

In addition, epidemiological studies suggested a negative correlation between alcohol intake, memory, cognition, and Alzheimer’s disease [71]. As ethanol negatively modulates NMDA receptors, Kalev-Zylinska and During hypothesized that moderate alcohol consumption could improve memory via adaptive responses in the expression of NMDA receptors and downstream signalling [71]. They were able to give experimental support to this hypothesis by working with rats bearing hippocampal knock-down of NMDA NR1 subunit and rats with increased hippocampal expression of NR1: they found that moderate ethanol intake improved memory, increased NR1 expression, and changed some aspects of neurotrophin signalling; NR1 knock-down prevented ethanol’s facilitatory effects; and hippocampal NR1 overexpression mimicked the effect of chronic low-dose ethanol intake on memory. On the other hand, high-dose ethanol reduced neurogenesis, inhibited NMDA NR2B subunit expression, and impaired visual memory. Then, it seems that the relationship between ethanol, memory, cognition, and Alzheimer’s disease might have a delicate dose-dependent balance.

The detrimental ethanol effect on colour vision that was observed in this work might be due do specific action of this xenobiotic on regions of the visual system that process colour information and are characterized by high energy consumption such as the central retina, layers 4A and 4Cb and the blobs of layers 2–3 of V1, as well as the thin strips of V2. It might be hypothesized that in these regions, the interplay between ethanol and neuronal function could go out of balance at lower doses than in other less demanding regions.

Some aspects of nervous system development proceed throughout adolescence and young adult age [72]. Thus, exposure to neurotoxicants during this period could have important noxious effects on visual system performance. In many countries, alcohol consumption is a problem for adolescents and young adults. We suggest that measurements of colour vision could be used to monitor the neural health of subjects belonging to this population.

## Supporting Information

S1 Dataset Spatial luminance contrast sensitivity of young alcohol consumers compared with tolerance limits for the control group. Colour arrangement performance of young alcohol consumers in the FM 100 test compared with performance of young non-alcohol consumers.
(XLSX)

S2 Dataset Colour discrimination ellipses from young alcohol consumers obtained with the MR test compared with young non-alcohol consumers. (XLSX)
